# BMSCs Regulate Astrocytes through TSG-6 to Protect the Blood-Brain Barrier after Subarachnoid Hemorrhage

**DOI:** 10.1155/2021/5522291

**Published:** 2021-06-29

**Authors:** Yilv Wan, Min Song, Xun Xie, Zhen Chen, Ziyun Gao, Xiang Wu, Rui Huang, Min Chen

**Affiliations:** ^1^Department of Neurosurgery, The First Affiliated Hospital of Nanchang University, Nanchang, Jiangxi 330000, China; ^2^The Second Clinical Medical College of Nanchang University, Nanchang, Jiangxi 330000, China; ^3^Department of Neurosurgery, The Second Affiliated Hospital of Nanchang University, Nanchang, Jiangxi 330000, China

## Abstract

**Background:**

In patients with subarachnoid hemorrhage (SAH), the damage of the blood-brain barrier (BBB) can be life-threatening. Mesenchymal stem cells are widely used in clinical research due to their pleiotropic properties. This study is aimed at exploring the effect of BMSCs regulating astrocytes on the BBB after SAH.

**Methods:**

The SAH model was established by perforating the blood vessels. BMSCs were transfected with TSG-6 inhibitor plasmid and cocultured with astrocytes. Intravenous transplantation of BMSCs was utilized to treat SAH rats. We performed ELISA, neurological scoring, Evans blue staining, NO measurement, immunofluorescence, BBB permeability, Western blot, HE staining, Nissl staining, and immunohistochemistry to evaluate the effect of BMSCs on astrocytes and BBB.

**Results:**

SAH rats showed BBB injury, increased BBB permeability, and brain histological damage. BMSCs will secrete TSG-6 after being activated by TNF-*α*. Under the influence of TSG-6, the NF-*κ*B and MAPK signaling pathways of astrocytes were inhibited. The expression of iNOS was reduced, while occludin, claudin 3, and ZO-1 expression was increased. The production of harmful substances NO and ONOO^−^ decreased. The level of inflammatory factors decreased. The apoptosis of astrocytes was weakened. TSG-6 secreted by BMSCs can relieve inflammation caused by SAH injury. The increase in BBB permeability of SAH rats was further reduced and the risk of rebleeding was reduced.

**Conclusion:**

BMSCs can regulate the activation of astrocytes through secreting TSG-6 in vivo and in vitro to protect BBB.

## 1. Introduction

Subarachnoid hemorrhage (SAH) is a devastating form of stroke that leads to incurable outcomes [1, 2]. When SAH occurs, a variety of pathophysiological reactions starts immediately. Early brain injury (EBI) refers to direct brain damage and secondary pathophysiological reactions within 72 hours after SAH occurs, including increased intracranial pressure and decreased cerebral blood flow, and the resulting local cerebral ischemia [3]. These changes immediately lead to neuronal cell death, blood-brain barrier (BBB) permeability changes, and brain edema [4–6]. In the basic structure that retains the normal function of the BBB, cytoplasmic adhesion proteins and transmembrane proteins together form its tight junction structure. Among them, cytoplasmic adhesion proteins include several subtypes of zonula occludens-1 (ZO-1), ZO-2, and ZO-3, which form the basis of tight junctions. Transmembrane proteins are comprised of occludin, claudin, junctional adhesion molecules (JAM), and tricellulin [7, 8]. Therefore, it can be determined whether the BBB is damaged by detecting cytoplasmic adhesion proteins and transmembrane proteins.

Astrocytes play an important role in maintaining the integrity of the BBB [9]. Astrocytes can produce large amounts of NO through inducible nitric oxide synthase (iNOS) under the action of some stimulating factors. NO combines with superoxide anion (O^2-^) to produce a metabolite called peroxynitrite (ONOO^−^). ONOO^−^ is not only a potent biological oxidant but also a very toxic and destructive product [10]. Our previous work confirmed that it can reduce the destruction of BBB and the degree of cerebral edema after cerebral hemorrhage by inhibiting the production of iNOS and ONOO^−^, but whether the target involves astrocytes is not unknown. Since astrocytes may have a close relationship with the amount of ONOO^−^ production in the state of 3neuroinflammation, bone marrow mesenchymal stem cells (BMSCs) were used to study the protective effect of BBB after SAH and astrocytes were used as the research target.

BMSCs are a group of adult stem cells with biological characteristics, multidifferentiation potential, and self-replication [11]. The recent literature review has noted that in SAH animal experiments, intravenous transplantation of BMSCs can improve the neurological function of SAH animals. The related mechanism may be linked to reducing neuronal apoptosis, reducing inflammation, and improving the microstructure of brain tissue. However, specific targets of BMSCs and the mechanisms involved need to be further studied [12]. More and more studies have confirmed that paracrine action played an important role in the treatment of BMSCs. Factors related to the paracrine action of BMSCs include interleukin 10 (IL-10), indoleamine 2,3-dioxygenase (indoleamine 2,3-dioxygenase), and tumor necrosis factor-induced protein 6 (TNF-*α*-induced protein 6 (TSG-6)) [13, 14]. TSG-6 has become an important anti-inflammatory factor for studying the paracrine effects of BMSCs [13, 15].

With the deepening of research on TSG-6, the downstream signaling pathways regulated by TSG-6 are gradually discovered by researchers. Choi has pointed out that TSG-6 had a bearing on the downstream nuclear factor kappa B (NF-*κ*B) signaling pathway [14]. In addition, related studies have believed that inhibiting the downstream NF-*κ*B signaling pathway was an important way for TSG-6 to exert its biological characteristics [16, 17]. The NF-*κ*B family includes transcription factors such as p65, which are involved in cell apoptosis and inflammation [18]. As another important pathway in the nervous system, mitogen-activated protein kinase (MAPK) is a set of proteases that can be stimulated by different cells and plays a role in regulating inflammation, oxidative stress, etc. [19]. The MAPK family includes p38MAPK, JNK, and ERK1/2. Among them, p38MAPK and JNK mainly play a role in inflammation and apoptosis, while ERK1/2 plays a role in proliferation and differentiation [20]. Although the results of previous studies supported that BMSCs can reduce brain edema after cerebral hemorrhage and protect BBB through TSG-6, there is no relevant research on whether BMSCs can decrease BBB damage through TSG-6 in SAH. In addition, since astrocytes play an important part in the neuroinflammatory process and maintaining the integrity of the BBB, whether TSG-6 regulates astrocyte targets through related signaling pathways needs further verification. Through the research of this subject, we can clarify the role of BMSCs in regulating target cells through TSG-6, to provide new ideas and basis for BMSCs in the research and treatment of SAH.

## 2. Results

### 2.1. Characteristic of SAH Modeling

**Figure 1:**
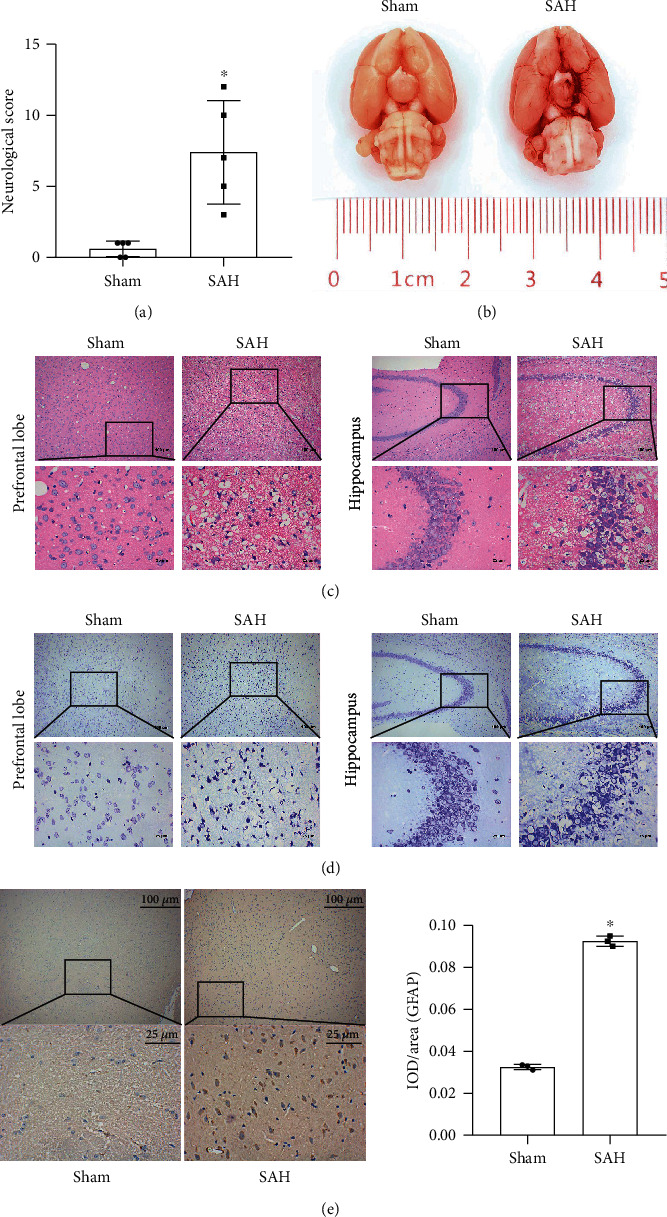
Pathological features of the SAH model. (a) Quantitative analysis of neurological scores. (b) Representative brains from the sham and SAH groups. (c) HE staining. (d) Nissl staining. (e) Immunohistochemical was used to detect of positive expression of activated astrocyte marker GFAP+. ^∗^Compared with the sham group, *P* < 0.05. The above results were all measurement data, expressed as mean ± SD, and an unpaired *t*-test was used between the two groups.

We established a rat model of SAH to observe the pathological changes of SAH. No rat died in the sham group (0 of 8 rats). Approximately, 13.51% of the rats (37 of 5) classified into the SAH group died within 24 hours after SAH induction. To observe whether the SAH model was successful, we performed the neurobehavioral score of mNSS on the rats. As found in Figure 1(a), the score of the sham group was significantly lower than that of the SAH group. After modeling, rats in the SAH group had walking instability, limb hemiplegia, and poor response to external stimuli. Subsequently, the brains of the two groups of rats were taken out. In Figure 1(b), we observed that the ventral surface of the rats in the sham group was smooth. No bleeding was seen, and the general structure of the brain tissues was not significantly changed. However, the SAH group rats showed obvious blood accumulation on the surface of the temporal cortex and no obvious changes in the gross structure of brain tissue. To further determine whether the model was successful, the degree of brain histological injury was observed by HE (Figure 1(c)) and Nissl staining (Figure 1(d)). HE staining showed histological damage in both the prefrontal lobe and hippocampus in the SAH group compared with the sham group. Nissl bodies were reduced and neurons were damaged in the prefrontal cortex and hippocampus in the SAH group compared with the sham group. Results of HE and Nissl staining showed that the neurologic scores obtained in this model were reliable and positively correlated with the histology/pathologic changes of the SAH brain. To further examine whether astrocytes were affected by the breakdown of the BBB, immunohistochemistry was used to measure the markers of activated astrocytes GFAP+.  Figure 1(e) revealed that GFAP+ positive was increased in the SAH group. The above suggested the success of SAH modeling. It was primarily manifested in the destruction of BBB and the activation of astrocytes.

### 2.2. BMSCs Secreted TSG-6 to Protect Activated Astrocytes

**Figure 2:**
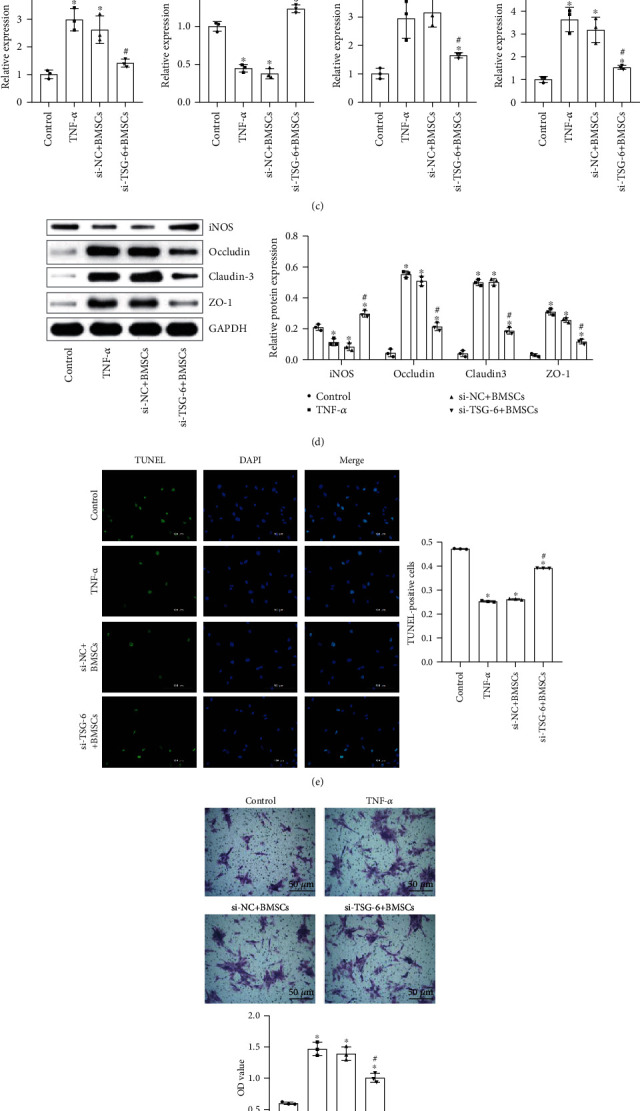
The effect of TSG-6 secreted by BMSCs on activated astrocytes. (a) qRT-PCR was utilized to detect TSG-6 expression in BMSC cells. (b) Western blot analysis of the expression of TSG-6 in BMSC cells. (c) qRT-PCR was utilized to test the expression levels of occludin, claudin 3, ZO-1, and iNOS in activated astrocytes. (d) Western blot was used to measure the protein expression levels of occludin, claudin 3, ZO-1, and iNOS in astrocytes. (e) TUNEL assay was used to detect astrocyte apoptosis (scale bar, 100 *μ*m). (f) Invasion of astrocytes was examined by transwell assay (scale bar, 50 *μ*m). BMSCs (medium containing BMSCs (TNF-*α* activation group)), si − NC + BMSCs (medium containing BMSCs (NC siRNA + TNF − *α* activation group)), and si − TSG − 6 + BMSCs (medium containing BMSCs (siRNA − TSG − 6 + TNF − *α* activation group)). ^∗^Compared with the control group, *P* < 0.05; ^#^compared with the si − NC + BMSC group, *P* < 0.05. The above experimental results were all measurement data. Data were expressed as the mean ± SD. One-way ANOVA and Tukey's test were used only for the data comparison between multiple groups.

To determine the effect of BMSCs on astrocytes in a SAH model, we conducted a series of in vitro experiments. In Figure 2(a), qRT-PCR was used to measure the expression of TSG-6. It was found that when TNF-*α* stimulated BMSC activation, the expression of TSG-6 increased. To study whether TSG-6 expression affected activated astrocytes, we silenced the TSG-6 gene of BMSC cells.  Figure 2(b) indicated that after silencing the TSG-6 gene, the expression level of TSG-6 decreased. It provided direct evidence that the silent TSG-6 was successfully constructed. Figures 2(c) and 2(d) data revealed that after BMSCs were stimulated by TNF-*α* to secrete TSG-6, TSG-6 promoted astrocyte occludin, claudin 3, and ZO-1 expression in both mRNA and protein levels, while iNOS was reduced. In the case of silencing TSG-6 in BMSCs, the expression levels of astrocyte occludin, claudin 3, and ZO-1 were reduced but iNOS was increased.  Figure 2(e) showed that BMSCs stimulated by TNF-*α* to secrete TSG-6 decreased astrocyte apoptosis while silencing of TSG-6 in BMSC promoted astrocyte apoptosis.  Figure 2(f) displayed that BMSCs stimulated by TNF-*α* to secrete TSG-6 increased the invasion ability of astrocytes while the invasion ability of astrocytes was reduced after silencing of TSG-6 in BMSCs. In summary, these findings suggested that TSG-6 secreted by BMSCs could increase the production of tight junction proteins in astrocytes, with increased invasion and less apoptosis.

### 2.3. BMSCs Regulated the NF-*κ*B/MAPK Signaling Pathway through TSG-6

**Figure 3:**
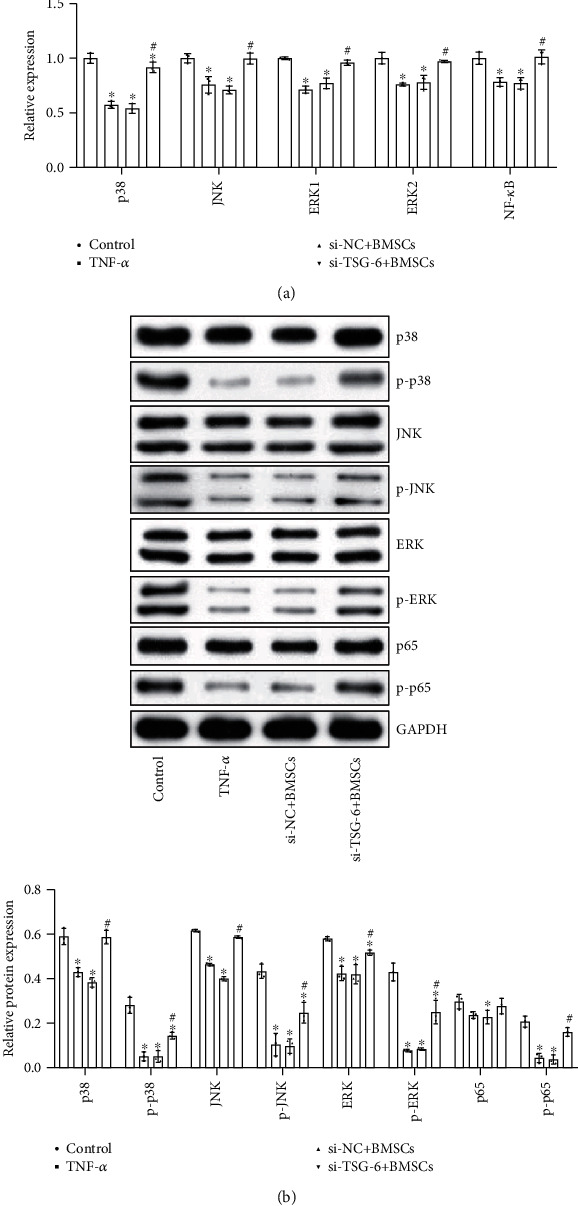
TSG-6 secreted by BMSCs inhibited the activation of the NF-*κ*B/MAPK signaling pathway. (a) qRT-PCR measured the expression of the NF-*κ*B/MAPK signaling pathway. (b) Western blot observed the protein expression of the NF-*κ*B/MAPK signaling pathway. BMSCs (medium containing BMSCs (TNF-*α* activation group)), si − NC + BMSCs (medium containing BMSCs (NC siRNA + TNF − *α* activation group)), and si − TSG − 6 + BMSCs (medium containing BMSCs (siRNA − TSG − 6 + TNF − *α* activation group)). ^∗^Compared with the control group, *P* < 0.05; ^#^compared with the si − NC + BMSC group, *P* < 0.05. The above experimental results were all measurement data. Data were expressed as the mean ± SD. One-way ANOVA and Tukey's test were used only for the data comparison between multiple groups.

We further investigated whether TSG-6 activated the NF-*κ*B/MAPK pathway in astrocytes. We used qRT-PCR and Western blot to measure the expression of the NF-*κ*B and MAPK signaling pathway genes. The data results in Figure 3 showed that compared with that in the control group, the expression of mRNAs related to the NF-*κ*B signaling pathway (NF-*κ*B) and MAPK signaling pathway (p38, JNK, ERK1, and ERK2) in the TNF-*α* group was inhibited. Compared with the si − NC + BMSC group, the expression of the NF-*κ*B signaling pathway (NF-*κ*B) and MAPK signaling pathway (p38, JNK, ERK1, and ERK2) in the si − TSG − 6 + BMSC group was considerably increased. The above results demonstrated that TSG-6 secreted by BMSCs could inhibit the NF-*κ*B/MAPK signaling pathway in astrocytes.

### 2.4. Astrocytes Were Protected by Inhibition of NF-*κ*B

**Figure 4:**
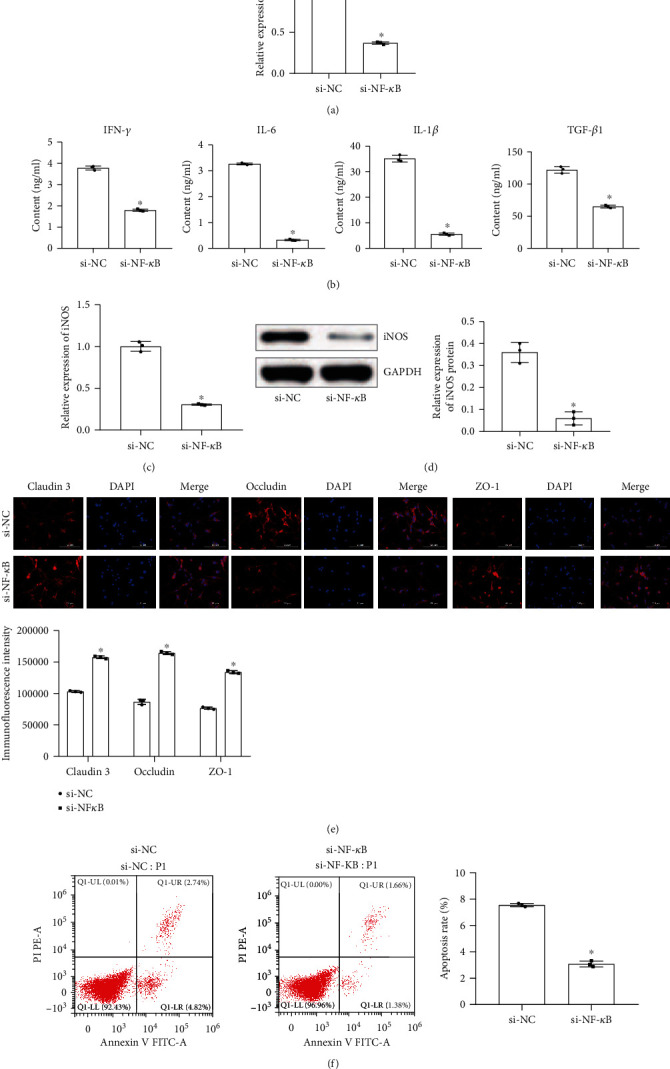
Astrocytes were protected by inhibition of NF-*κ*B. (a) qRT-PCR tested the expression of NF-*κ*B. (b) ELISA was used to measure the content of IFN-*γ*, IL-6, IL-1*β*, and TGF-*β*1 in astrocytes. (c) qRT-PCR detected the expression of iNOS. (d) Western blot measured the protein expression of iNOS. (e) Fluorescence intensity of claudin 3, occludin, and ZO-1 was detected by immunofluorescence (scale bar, 100 *μ*m). (f) Flow cytometry was used to measure the apoptosis rate of astrocytes. ^∗^Compared with the si-NC group, *P* < 0.05. The above experimental results were all measurement data. Data are expressed as the mean ± SD. The unpaired *t*-test was used between the two groups.

The above experimental results suggested that TSG-6 secreted by BMSCs can inhibit the expression of the NF-*κ*B signaling pathway. We further studied the effect of the NF-*κ*B signaling pathway on activating astrocytes. First, we used the NF-*κ*B blocker to silence the expression of NF-*κ*B in astrocytes.  Figure 4(a) demonstrated that the expression of NF-*κ*B in the si-NF-*κ*B group decreased, indicating that the si-NF-*κ*B group was successfully constructed. Data in Figures 4(b)–4(d) showed that compared with the si-NC group, the levels of related inflammatory factors IL-6, IL-1*β*, IFN-*γ*, TGF-*β*1, and iNOS in the si-NF-*κ*B group decreased. This indicated that si-NF-*κ*B reduced the inflammatory response in astrocytes.  Figure 4(e) showed that si-NF-*κ*B increased occludin, claudin 3, and ZO-1 expression in astrocytes. We further detected astrocyte apoptosis.  Figure 4(e) showed that si-NF-*κ*B inhibited astrocyte apoptosis. Therefore, inhibition of the NF-*κ*B signaling pathway was an effective way to protect astrocytes against injury. We speculated that TSG-6 secreted by BMSCs may protect activated astrocytes by inhibiting the expression of the NF-*κ*B signaling pathway.

### 2.5. Inhibition of the MAPK Signaling Pathway Protected Astrocytes

**Figure 5:**
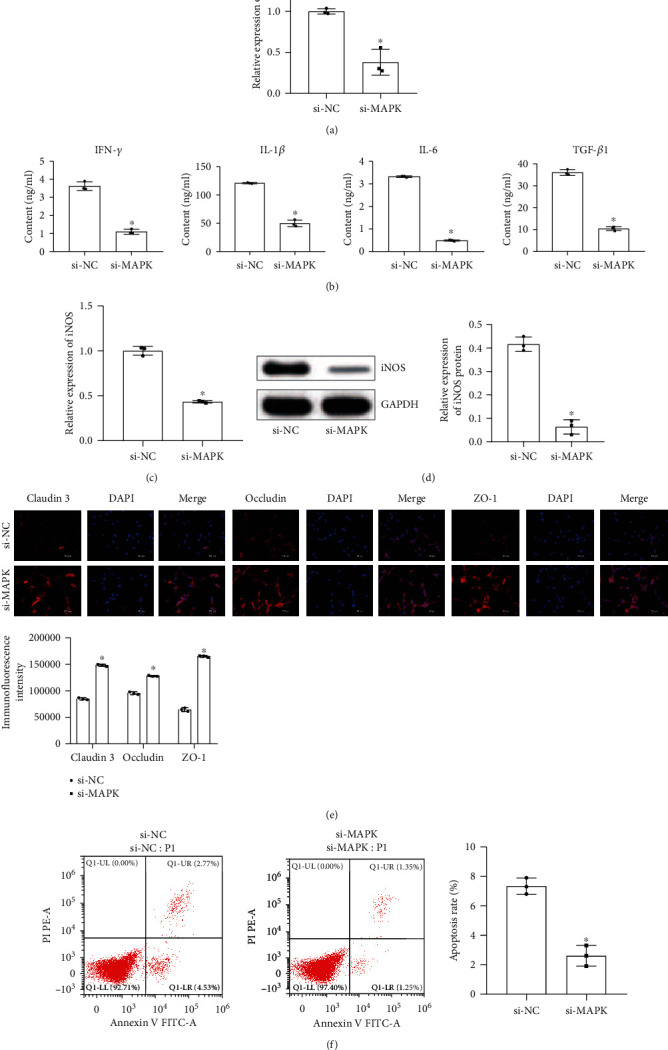
Inhibition of the MAPK signaling pathway to regulate astrocytes. (a) qRT-PCR detected p38 expression. (b) ELISA to measure the contents of IFN-*γ*, IL-1*β*, IL-6, and TGF-*β*1. (c) qRT-PCR examined the expression of iNOS. (d) Western Blot examined the protein expression of iNOS. (e) Immunofluorescence detection of occludin, claudin 3, and ZO-1 fluorescence intensity (scale bar, 100 *μ*m). (f) Flow cytometry was used to measure the apoptosis rate of astrocytes. ^∗^Compared with the si-NC group, *P* < 0.05. The above experimental results were all measurement data. Data are expressed as the mean ± SD. The unpaired *t*-test was used between the two groups.

To learn the influence of the MAPK signaling pathway on astrocytes, we primarily used MAPK blockers to silence the expression of MAPK in astrocytes. The result of Figure 5(a) revealed that the expression of p38 decreased, indicating that the si-MAPK group was successfully constructed. Figures 5(b)–5(f) showed that after inhibiting the expression of MAPK, the levels of related inflammatory factors IL-6, IL-1*β*, IFN-*γ*, TGF-*β*1, and iNOS secreted by astrocytes decreased. Moreover, the fluorescence intensity of occludin, claudin 3, and ZO-1 increased. Finally, the flow cytometry results indicated that astrocyte apoptosis was reduced in the si-MAPK group compared with the si-NC group. In short, inhibiting the MAPK signaling pathway could also protect activated astrocytes. We suspected that TSG-6 secreted by BMSCs may protect astrocytes by inhibiting the expression of the MAPK signaling pathway.

### 2.6. BMSCs Regulated Astrocytes through TSG-6 to Protect the BBB after SAH

**Figure 6:**
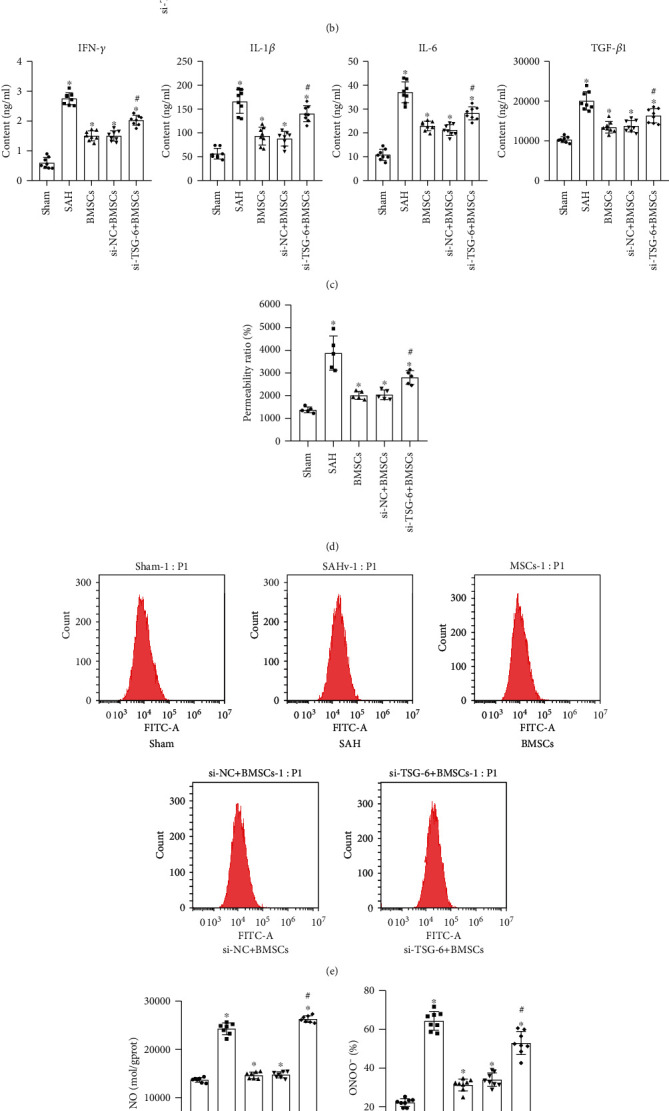
BMSCs regulate astrocytes through TSG-6 to treat rats suffering from SAH. (a) The expression of TSG-6 and iNOS was measured by qRT-PCR and Western blot. (b) qRT-PCR and Western blot were used to measure the expression level of the NF-*κ*B/MAPK signaling pathway. (c) ELISA was used to test the level of inflammatory factors secreted by activated astrocytes. (d) Evans blue detected changes in BBB permeability. (e) NO fluorescent probe analyzed the amount of NO production in the cell. (f) Determination of the ONOO^−^ level in rat serum by ELISA. (g) Double immunofluorescence staining was used to measure the expression of iNOS (red) and GAFP (green). BMSCs (medium containing BMSCs (TNF-*α* activation group)), si − NC + BMSCs (medium containing BMSCs (NC siRNA + TNF − *α* activation group)), and si − TSG − 6 + BMSCs (medium containing BMSCs (siRNA − TSG − 6 + TNF − *α* activation group)). ^∗^Compared with the sham group, *P* < 0.05; ^#^compared with the si − NC + BMSC group, *P* < 0.05. The above experimental results were all measurement data. Data are expressed as the mean ± SD. One-way ANOVA and Tukey's test were used for the data comparison between multiple groups.

To further study whether TSG can protect the BBB, we conducted animal experiments to explore.  Figure 6(a) examined that the SAH model resulted in decreased TSG-6 and increased iNOS expression in brain tissue, while activated BMSCs promoted TSG-6 and reduced iNOS protein expression. Silencing of TSG-6 in BMSCs can decrease TSG-6 and promote iNOS protein expression. Compared with the si − NC + BMSC group, the expression of p38, JNK, p-JNK, ERK, p-ERK, p65, and p-p65 was increased in the si − TSG − 6 + BMSC group. The data in Figure 6(b) showed that inhibition of TSG-6 can promote the expression of genes associated with the NF-*κ*B/MAPK signaling pathway. Compared with the si − NC + BMSC group, the levels of IFN-*γ*, IL-6, IL-1*β*, and TGF-*β*1 in the si − TSG − 6 + BMSC group were increased. It was found in Figure 6(c) that the inflammatory response of astrocytes is enhanced after blocking the expression of TSG-6 in BMSCs. In Figure 6(d), Evans blue staining was used to measure the permeability of the BBB. The results demonstrated that silencing of TSG-6 in BMSCs can enhance the permeability of the BBB, which caused harmful substances to enter the neurons of the brain tissue. Figures 6(e) and 6(f) identified that TSG-6 secreted by BMSCs can reduce the formation of NO and ONOO^−^ in SAH rats which is a direct result of BBB destruction. In Figure 6(g), the immunofluorescence results showed that TSG-6 secreted by BMSCs downregulated the expression of GFAP and iNOS in SAH rats, while the expression of GFAP and iNOS was upregulated in the si − TSG − 6 + BMSC group compared with the si − NC + BMSC group. The above indicated that TSG-6 secreted by BMSCs could inhibit inflammation and decrease BBB damage in SAH rats through the NF-*κ*B/MAPK signaling pathway.

## 3. Material and Methods

### 3.1. Animals

We purchased 45 SPF (specific pathogen-free) SD rats with a weight of 200 ± 20 g and 6–8 weeks old from Hunan Slack Jingda Experimental Animal Co. Ltd. The disposal of animals during the experiment complied with the “Guiding Opinions on Treating Experiment Animals” issued by the Ministry of Science and Technology in 2006. Eight of them were selected as the sham group, and the remaining 37 were used to construct the SAH model.

### 3.2. SAH Model

The SAH model was established by perforating the blood vessels. The animals were anesthetized by intraperitoneal injection of 1% (*w*/*v*) sodium pentobarbital (35 *μ*mg/kg). The bifurcation of the right common carotid artery was exposed. The external carotid artery was separated. The blood flow was blocked with a vascular clamp. Blunt 3-0 single-stranded nylon sutures were inserted into the internal carotid artery from the bifurcation of the common carotid artery 18–20 mm to cause the artery to rupture. The suture was maintained for about 15 s and then pulled out. Then, the wound was closed. The procedure of the sham group was as of the SAH group, except that the blood vessel was not punctured. 37 animals were used for modeling, of which 5 died. The modeling success rate was calculated by the number of animals successfully modeled/total number of animals (the modeling success rate = 32/37∗100%).

### 3.3. Cell Culture

Human bone marrow mesenchymal stem cells (hBMSCs) and astrocytes were purchased from HonorGene (Changsha Aibiwei Biotechnology Co. Ltd.). 2 × 10^5^ astrocytes/well were activated by lipopolysaccharide (LPS) and placed in the lower transwell compartment. BMSCs were cultured in the human mesenchymal stem cell culture medium (Cyagen Biosciences Inc., Guangzhou, China) at 37°C and 5% CO_2_. When the third-generation BMSC reached 80% fusion, which was inoculated in a 24-well culture plate at 1.0 × 10^5^ cells/well, 2 mL of complete medium was added to each well. BMSCs are activated by TNF-*α*. TSG-6 siRNA and NC siRNA were taken out and frozen on ice. Two centrifuge tubes were taken, and each tube was added with a 95 *μ*L serum-free medium, and then, 100 *μ*g TSG-6 siRNA and 5 *μ*L Lip 2000 were added into the centrifuge tubes, respectively. NC siRNA was also added to the corresponding centrifuge tubes in this way. TSG-6 siRNA and NC siRNA were transfected into BMSCs activated by TNF-*α*. The cells were then cultured at 37°C for 24 hours. Subsequently, BMSCs of one of the above treatments were placed in the upper compartment and cocultured with astrocytes at 37°C for 24 h. According to the composition contained in the medium of the upper chamber, they were divided into control (medium-control group), BMSCs (medium containing BMSCs (TNF-*α* activation group)), si − NC + BMSCs (medium containing BMSCs (NC siRNA + TNF − *α* activation group)), and si − TSG − 6 + BMSCs (medium containing BMSCs (siRNA − TSG − 6 + TNF − *α* activation group)).

### 3.4. BMSC Intervention In Vivo

3 × 10^6^ cells (including BMSCs, si − NC + BMSCs, and si − TSG − 6 + BMSCs) in what was the same treatment as in the cell experiment were suspended in 1 mL of PBS within 1 hour after successful induction of SAH and then slowly injected through the femoral vein for 5 minutes. The needle was pulled out. The femoral vein was ligated. In the SAH group, rats were intravenously injected with the same amount of PBS without BMSCs. Then, the 32 successfully modeled animals were divided into 5 groups, 8 in each group: the SAH group, BMSC group, si − NC + BMSC group, and si − TSG − 6 + BMSC group.

### 3.5. Assessment of Neurological Injury

The modified neurological severity score (mNSS) includes a series of comprehensive tests to evaluate motor (muscle state, abnormal movement), sensory (visual, tactile, and proprioceptive), and reflex capabilities. A point was awarded for a specific task or tested reflexes that were not performing. Therefore, the higher the score, the more serious the injury (normal score: 0; maximum score: 18). After successful modeling, mNSS was evaluated to determine the severity of the injury.

### 3.6. Enzyme-Linked Immunosorbent Assay (ELISA)

After the specimens were placed at room temperature for 2 hours, they were centrifuged at 2–8°C at 1000 g for 15 minutes and the supernatant was taken for testing. The kit was as follows: IL-1*β* (China, CSB-E04640R), IL-6 (China, CSB-E08055R), IFN-*γ* (China, CSB-E04579R), and TGF-*β*1 (China, CSB-E04727R) were purchased from Cusabio Biotech Co. Ltd. ONOO^−^ (China, JL21035) was purchased from Jianglaibio Co. Ltd. Horseradish peroxidase-labeled avidin solution 100 *μ*L was added to each well and incubated at 37°C for 1 hour. Substrate solution (90 *μ*L) was added to each well in order, and color was developed at 37°C for 15–30 minutes. 50 *μ*L of termination solution was added to each well in sequence to terminate the reaction. Within 5 minutes after the end of the reaction, the optical density (OD value) of each well was measured sequentially under an enzyme standard instrument (China, MB-530) at the wavelength of 450 nm.

### 3.7. Quantitative Real-Time PCR (qRT-PCR)

The total RNA from tissue and cell samples was extracted using a TRIzol kit (15596026, Thermo, USA). In the next step, the extracted total RNA was reversely transcribed into cDNA according to the instruction of a reverse transcription kit (CW2569, CWBIO, China). Subsequently, real-time PCR was performed on a fluorescence quantitative RCP instrument (QuantStudio 1, Thermo, USA) using an UltraSYBR Mixture (CW2601, CWBIO, China). The internal reference was *β*-actin, and the primer sequence was found in Table 1. With 2 *μ*g cDNA as the template, the relative quantitative method (2^−△△*Ct*^ method) was used to calculate the relative transcription level of the target gene: △△*Ct* = △ experimental group −△ control group,△*Ct* = *Ct*(target gene) − *Ct*(*β* − actin). The experiment was repeated three times.

### 3.8. Western Blot

The RIPA kit (R0010, Solarbio, China) was used to extract the total protein of tissue and cell samples. The BCA method was used to determine the protein concentration. The protein was separated by 10% SDS-PAGE electrophoresis and transferred to the NC membrane by electrotransfer. The membrane was blocked with 5% skimmed milk for 2 h at room temperature and incubated with the primary antibody overnight at 4°C. For primary antibodies, we used rabbit anti-TSG-6 (0.1 *μ*g/mL, ab204049, Abcam), rabbit anti-iNOS (1 : 500, ab3523, Abcam), rabbit anti-ZO-1 (1 : 4000, 21773-1-AP, Proteintech), rabbit anti-occludin (1 : 1000, 27260-1-AP, Proteintech), rabbit anti-claudin 3 (1 : 500, 16456-1-AP, Proteintech), rabbit anti-p38 (1 : 1000, 14064-1-AP, Proteintech), rabbit anti-p-p38(1 : 1000, ab4822, Abcam), rabbit anti-JNK (1 : 1000, ab179461, Abcam), rabbit anti-p-JNK (1 : 5000, ab124956, Abcam), rabbit anti-p65 (1 : 800, 10745-1-AP, Proteintech), rabbit anti-p-p65 (1 : 800, ab97726, Abcam), rabbit anti-ERK (1 : 2000, 16443-1-AP, Proteintech), rabbit anti-p-ERK (1 : 5000, ab201015, Abcam), and rabbit anti-GAPDH (1 : 5000, 10494-1-AP, Proteintech). The membrane was rinsed 3 times with TBST for 10 minutes each time and then incubated with HRP goat anti-rabbit IgG (1 : 5000, SA00001-2, Proteintech, USA). The membrane was immersed in Super ECL Plus (K-12045-D50, Advansta, USA) for luminescence development. GAPDH was used as an internal reference. The target band was analyzed by ImageJ software.

### 3.9. Transwell Assay

MSC cells were cultured in DMEM containing 10% FBS with 1% double resistance and NESS in an incubator at 37°C, 5% CO_2_, and saturated humidity. Logarithmically grown cells were placed in a six-well plate and treated in groups after adherence to the wall. Matrigel was diluted with 100 *μ*L cold, serum-free MEM medium for each well, and the final concentration was 200 *μ*g per well. Matrigel was incubated at 37°C for 30 minutes and the supernatant was sucked out. 500 *μ*L complete medium of 10% FBS was placed in the lower compartment at 37°C for 48 h. The upper chamber was taken out and put into a new well containing PBS. The upper chamber was washed with PBS 3 times, and the upper chamber cells were wiped clean with a cotton ball. The cell was fixed with 4% paraformaldehyde for 20 minutes. 0.1% crystal violet was dyed for 5 min and washed 5 times. The film was given to the slide and photographs were taken under the microscope. The chamber was taken out and soaked in 10% acetic acid for decolorization. At 550 nm, the absorbance (OD) value was determined by a microplate reader.

### 3.10. Flow Cytometry

The cells were washed with PBS twice and centrifuged at 2000 rpm for 5 min each. About 1–5 × 10^5^ cells were collected. 500 *μ*L binding buffer was in addition to suspend cells. After Annexin V-FITC (5 *μ*L) was added, 5 *μ*L propidium iodide was added and mixed. The reaction lasted for 5–15 min at room temperature and away from light. The results were noted and detected by flow cytometry within 1 h.

### 3.11. TUNEL

The cell climbing piece was fixed with 4% paraformaldehyde for 30 minutes. Proteinase K working fluid was prepared. 100 *μ*L 1 × equilibrium buffer was added to each sample and incubated at room temperature for 10–30 min. 50 *μ*L TdT incubation buffer was added to the cell climbing piece. The DAPI (Wellbio, China) working fluid was dyed at 37°C for 10 min. The cell climbing piece was washed with PBS 5 min 3 times. The samples were stored in the dark and observed under a fluorescence microscope (BA410T, Motic, China).

### 3.12. Immunohistochemistry (IHC)

The slices were baked at 60°C for 12 h. The slices were dewaxed and heated to repair the antigen. An appropriately diluted primary antibody GFAP (556330, BD Pharmingen) was added overnight at 4°C. 50–100 *μ*L anti-mouse IgG-HRP (Bio-Rad, Hercules, CA) was added and incubated at 37°C for 30 min and then washed with PBS for 5 min 3 times. Diaminobenzidine (DAB, ZSGB-BIO, China) was used to be a chromogen. The samples were placed in xylene for 10 min 2 times. The neutral gum was sealed and enforced under a microscope.

### 3.13. Immunofluorescence (IF)

We took out the cell climbing piece and washed it with PBS 2–3 times. 0.3% Triton was added at 37°C for 30 minutes. The slices were baked at 60°C for 12 h. The slices were dewaxed and heated to repair the antigen. The slices were incubated with 5% BSA at 37°C for 60 minutes. Primary antibody claudin 3 (1 : 50, 16456-1-AP, Proteintech), occludin (1 : 50, 27260-1-AP, Proteintech), and ZO-1 (1 : 200, 21773-1-AP, Proteintech) were added to the cell climbing pieces at 4°C overnight. 50–100 *μ*L anti-rabbit IgG-labeled fluorescent antibodies (1 : 200, SA00013-4, Proteintech) were added to the cell climbing pieces and incubated at 37°C for 90 min. Primary antibody GFAP (1 : 1000, ab279290, Proteintech) and iNOS (1 : 500, ab3523, Proteintech) were added to the slices at 4°C overnight. 50–100 *μ*L anti-mouse (1 : 200, SA00013-5, Proteintech) and rabbit (1 : 200, SA00013-4, Proteintech) IgG-labeled fluorescent antibodies were added to the cell climbing pieces and incubated at 37°C for 90 min. DAPI solution was used to stain the nucleus at 37°C for 10 min, and PBS was used to wash the cell climbing piece for 5 min 3 times. Buffer glycerin was used to seal the cell climbing piece. The samples were stored in the dark and observed under a fluorescence microscope (BA210T, Motic, China).

### 3.14. Evans Blue Dye Assays

Rats were injected intravenously with 8 mL/kg 0.6% NaF 2 hours after successful modeling. After 30 minutes, the rats were perfused with 10 mL normal saline. The cerebral hemispheres were homogenized with 30% trichloroacetic acid and centrifuged at 10000 g for 5 minutes. From the supernatant, the concentration of NaF was measured with a fluorometer under excitation at 460 nm and emission at 515 nm through a standard curve. The permeability ratio was calculated by dividing the NaF (pg/mg) of the study case/the NaF of the normal control.

### 3.15. Assay of Intracellular NO Production

Cells were resuspended with diluted DAF-FM DA at a concentration of 1 × 10^6^/mL. The cells were incubated in a 37°C cell incubator for 20 minutes, and mixed upside down every 5 minutes to make the probe fully contact with the cells. The cells were washed three times with PBS (pH 7.4) to sufficiently remove DAF-FM DA that did not enter the cells. Fluorescence intensity was measured by a flow meter under 495 nm excitation wavelength and 515 nm emission wavelength.

### 3.16. HE Staining

The brain tissue was fixed with 4% paraformaldehyde at room temperature for 24 h. The samples were dehydrated in gradient ethanol and embedded into a wax block. Each sample was cut into 2–3 *μ*m slices. The slices were baked at 62°C for 2–6 h, then dewaxed, and rehydrated. The cytoplasm was stained with different degrees of red and or pink, in sharp contrast with the blue nucleus. The slices were observed under a microscope (BA210T, Motic, Singapore).

### 3.17. Nissl Staining

The brain tissue was fixed with 4% paraformaldehyde at room temperature for 24 h. The samples were dehydrated in gradient ethanol and embedded into a wax block. Each sample was cut into 2–3 *μ*m slices. The slices were baked at 62°C for 2–6 h, then dewaxed, and rehydrated. The slices were added with Nissl staining solution and incubated for 5 min. The slices were added with Nissl differentiation solution until nuclei and particles were clear. The slices were observed under a microscope (BA210T, Motic, Singapore).

### 3.18. Statistical Analysis

All Data were analyzed by GraphPad Prism 8.0 software (GraphPad Software, San Diego, California, USA). The unpaired *t*-test was used between the two groups conforming to the normal distribution. Comparisons among multiple groups were conducted by one-way analysis of variance (ANOVA), followed by Tukey's post hoc test. A value of *P* < 0.05 was considered to be statistically significant.

## 4. Discussion

The purpose of this study was to determine whether TSG-6 secreted by BMSCs could protect the BBB after SAH by regulating the NF-*κ*B/MAPK signaling pathway. To this end, we discussed in vitro and in vivo levels. In vitro experiments showed that TSG-6 secreted by BMSCs upregulated the expression of ZO-1, occludin, and claudin 3, downregulated the expression of iNOS and cell apoptosis, promoted cell invasion, and inhibited the NF-*κ*B/MAPK signaling pathway in astrocytes. Silencing TSG-6 in BMSCs canceled this effect in astrocytes. Further inhibition of the NF-*κ*B/MAPK signaling pathway showed that the inflammatory response of astrocytes was decreased, the expressions of ZO-1, occludin, and claudin 3 were upregulated and apoptosis was inhibited. In addition, in vivo studies confirmed that transplanted BMSCs can effectively protect BBB by secreting TSG-6.

Currently, early brain injury (EBI) and delayed cerebral ischemia (DCI) are the main manifestations of SAH in two stages. Neuroprotection and antivasospasm are the most studied directions in many studies [21, 22]. Once the intracranial aneurysm ruptures, SAH is usually catastrophic because there is no effective treatment for the combined brain injury [23]. In most countries, nimodipine is the only drug approved for the treatment of SAH because no other intervention has been shown to work [24]. Therefore, there is an urgent need to study potential molecular targets of SAH to optimize therapeutic efficacy. In recent years, the research on BMSC transplantation is very hot. Predecessors verified the effectiveness and safety of BMSCs in the treatment of traumatic brain injury in mice [25]. We targeted astrocytes, which are closely related to the BBB, to treat SAH by intravenous transplantation of BMSCs. The endovascular perforation model of SAH is indeed not reproducible. In recent years, the endovascular perforation model duplicated the early pathophysiology of SAH. It has been widely used to study the early brain injury after SAH [26–29]. The endovascular perforation model has been proposed for clinical trial [30]. Therefore, in this project, we constructed an endovascular perforation rat model to study the effects of BMSCs on astrocytes and BBB after SAH through secreting TSG-6. After BMSCs activated by TNF-*α* were transplanted through the vein, BMSCs secreted a large amount of TSG-6. We found that TSG-6 could regulate the activation of astrocytes, protect BBB, and effectively treat SAH.

BBB destruction and neuroinflammation are the main pathological changes of brain injury, which lead to poor prognosis after SAH [31]. Studies have confirmed that TSG-6 is a powerful anti-inflammatory factor. Its powerful anti-inflammatory effects mainly include inhibition of proteases in the inflammatory network, binding to corresponding hydrophobic acid fragments and blocking the proinflammatory effects of the bound and inhibiting neutrophils to migrate to the center of inflammation [32]. Studies have also established a rat brain trauma model to confirm that a large amount of paracrine TSG-6 after intravenous transplantation of BMSCs can improve the neurological dysfunction of rats [33]. Previous studies mainly revealed that exogenous TSG-6 exhibited anti-inflammatory properties. We conducted experiments on this basis, and the results were in line. The focus of our research was placed on targeting TSG-6 to regulate astrocytes in the SAH rat model. Our data showed that the expression of TSG-6 in brain tissue was decreased after SAH. TSG-6 secreted by BMSCs promoted the expression of ZO-1, occludin, and claudin 3 in astrocytes, decreased the expression of iNOS and apoptosis level, promoted cell invasion, protected BBB, and alleviated early brain injury. Astrocyte injury and early brain injury were aggravated to some extent after TSG-6 was silenced in BMSCs. These results indicated that the neuroprotective effect of BMSCs was related to phenotypic regulation of astrocyte translocation through TSG-6. But the way of action was not clear yet, which is the content of our next research. Considering the limited budget and space, we did not evaluate neurobehavior dysfunction after treatment of BMSC and si − TSG − 6 + BMSC in this project. In future research, we will further improve. It is necessary to indicate where the BMSCs go in the brain and whether they can release TSG-6 in the SAH brain. It can further confirm in vivo whether BMSCs act on astrocytes in the brain and whether TSG-6 was secreted by BMSCs to protect BBB after SAH. We hypothesized that BMSCs protected BBB by secreting TSG-6 in some form. In the next project, we will explore this conjecture through fluorescence labeling in vivo and in vitro. Results need to be confirmed by using the TSG-6 KO mice to exclude the effect of endogenous TSG-6. Due to limited funding and inadequate animal handling facilities, TSG-6 KO was not used in this study to exclude endogenous TSG-6 interference. We will improve in the following study. This project lays a solid foundation for the following research.

Many important molecules participate in the destruction of the BBB through various independent or related signal transduction pathways. NF-*κ*B is a key transcription factor regulating the expression of various proinflammatory genes [34]. To elucidate the molecular mechanism of TSG-6 anti-inflammatory function, we first evaluated its effect on NF-*κ*B activity. The results revealed that TSG-6 secreted by BMSCs inhibited the expression of p65 and p-p65 in LPS-activated astrocytes and the inflammatory response was alleviated. Silencing TSG-6 in BMSCs had the opposite effect. In addition to the NF-*κ*B pathway, it has also been observed that MAPK can mediate the expression of GFAP and various astrocyte regulatory molecules in vivo and in vitro [35]. The MAPK pathway may be triggered by oxidative stress, which may hinder the recovery of the BBB. This may be linked to the increase in the concentration of proinflammatory cytokines and mediators (IFN-*γ*, IL-6, and IL-1*β*). Our results showed that TSG-6 secreted by BMSCs inhibited LPS-activated astrocytes p-38, p-p38, JNK, p-JNK, ERK, and p-ERK levels and reduced the inflammatory response. Silencing TSG-6 in BMSCs promoted the expression of p-38, p-p38, JNK, p-JNK, ERK, p-ERK, and inflammatory response. The results in vivo were consistent with those in cells. We hypothesized that TSG-6 secreted by BMSCs protected astrocytes and BBB by inhibiting the NF-*κ*B/MAPK signaling pathway. To test this conjecture, we further inhibited the NF-*κ*B/MAPK signaling pathway in astrocytes. We found that the inflammatory response of astrocytes was reduced. The expressions of ZO-1, occludin, and claudin 3 were upregulated. Apoptosis was inhibited. Considering the limitations of space and budget, we did not perform inhibition of the NF-*κ*B/MAPK signaling pathway in vivo to observe the effect of transplanted BMSCs on BBB of SAH rats by secreting TSG-6. Due to the small sample size, we did not carry out the analysis of sample size estimation, which needs to be further improved. In future projects, we will further explore the potential mechanism by which transplanted BMSCs protect BBB in SAH rats by secreting TSG-6. We are also interested in whether inflammatory factors affect the BBB in a concentration-dependent manner, and we have further research ideas on the peculiar ways of their regulation. In our future research, we will pay more attention to the treatment of SAH by clinical vein transplantation of BMSCs.

## 5. Conclusion

In conclusion, our data demonstrated that TSG-6 secreted by BMSCs could play endogenous brain protection after SAH. In vitro, we demonstrated that TSG-6 secreted from BMSCs can induce astrocytes to anti-inflammatory through the NF-*κ*B/MAPK pathway. In vivo, we demonstrated that BMSCs regulated the activation of astrocytes through secreting TSG-6 to protect BBB after SAH. Our findings may provide new ideas and basis for BMSCs in the research and treatment of SAH.

## Figures and Tables

**Table 1:** Primer sequences.

Gene	Sequences (5′-3′)
TSG-6	F: TCTTTACAGACCCGAAGCG
	R: TCTTCCTACAAAGCCGTGGAC
iNOS	F: AGGCACAAGACTCTGACACCC
	R: CGCACTTCTGTCTCTCCAAACCC
Occludin	F: CCCAGACCACTATGAAACCGACT
	R: CAGCCATGTACTCTTCGCTCT
Claudin 3	F: AAGATGTACGACTCGCTGCT
	R: CTGCCAGTAGGATAGACACCAC
p38	F: AGCTTACCGATGACCACGTT
	R: CACGTAGCCGGTCATTTCGTC
JNK	F: ACGAGTTTTATGATGACGCCTT
	R: CCACAGACCATAAATCCACGTT
ERK1	F: GCTGCTGTGTCTTTATCTATCCC
	R: CTCCACCCCTCTGTAGCAC
ERK2	F: TCATTCGCTGCAAGATGGAC
	R: TAAATCCCCAGGCAGTGAGCAT
RELA	F: CACCAAAGACCCACCTCACCG
	R: CTTGCTCCAGGTCTCGCTTC
ZO-1	F: CCTAATAAGAACAGAGCCGAGCA
	R: GCAACATCAGCAATCGGTCCA
*β*-Actin	F: ACATCCGTAAAGACCTCTATGCC
	R: TACTCCTGCTTGCTGATCCAC

## Data Availability

The data used to support to the findings of this study are available from the corresponding author upon request.
